# Novel non-stimulants rescue hyperactive phenotype in an *adgrl3.1* mutant zebrafish model of ADHD

**DOI:** 10.1038/s41386-022-01505-z

**Published:** 2022-11-18

**Authors:** Hildur Sóley Sveinsdóttir, Christian Christensen, Haraldur Þorsteinsson, Perrine Lavalou, Matthew O. Parker, Alena Shkumatava, William H. J. Norton, Emile Andriambeloson, Stéphanie Wagner, Karl Ægir Karlsson

**Affiliations:** 1 3Z, Reykjavik, Iceland; 2 Institut Curie, PSL Research University, INSERM U934, CNRS UMR 3215, F-75005 Paris, France; 3 School of Pharmacy and Biomedical Science, University of Portsmouth, Portsmouth, UK; 4 Department of Genetics and Genome Biology, University of Leicester, Leicester, LE1 7RH UK; 5 Neurofit SAS, Illkirch, France; 6 School of Science and Engineering, Reykjavik University, Reykjavik, Iceland; 7 Biomedical Center, University of Iceland, Reykjavik, Iceland

**Keywords:** Drug screening, Mutation

## Abstract

ADHD is a highly prevalent neurodevelopmental disorder. The first-line therapeutic for ADHD, methylphenidate, can cause serious side effects including weight loss, insomnia, and hypertension. Therefore, the development of non-stimulant-based therapeutics has been prioritized. However, many of these also cause other effects, most notably somnolence. Here, we have used a uniquely powerful genetic model and unbiased drug screen to identify novel ADHD non-stimulant therapeutics. We first found that *adgrl3.1* null (*adgrl3.1*^−/−^) zebrafish larvae showed a robust hyperactive phenotype. Although the hyperactivity was rescued by three ADHD non-stimulant therapeutics, all interfered significantly with sleep. Second, we used wild-type zebrafish larvae to characterize a simple behavioral phenotype generated by atomoxetine and screened the 1200 compound Prestwick Chemical Library® for a matching behavioral profile resulting in 67 hits. These hits were re-assayed in the *adgrl3.1*^−/−^. Using the previously identified non-stimulants as a positive control, we identified four compounds that matched the effect of atomoxetine: aceclofenac, amlodipine, doxazosin, and moxonidine. We additionally demonstrated cognitive effects of moxonidine in mice using a T-maze spontaneous alternation task. Moxonidine, has high affinity for imidazoline 1 receptors. We, therefore, assayed a pure imidazoline 1 agonist, LNP599, which generated an effect closely matching other non-stimulant ADHD therapeutics suggesting a role for this receptor system in ADHD. In summary, we introduce a genetic model of ADHD in zebrafish and identify five putative therapeutics. The findings offer a novel tool for understanding the neural circuits of ADHD, suggest a novel mechanism for its etiology, and identify novel therapeutics.

## Introduction

Attention-deficit/hyperactivity disorder (ADHD) is a neurodevelopmental disorder with a prevalence of approximately 2–5% worldwide [1]. Symptoms appear as early as in preschool, with some patients showing pervasive behavioral and psychiatric deficits into adulthood [2]. ADHD is often associated with functional and psychosocial comorbidities as well as a higher risk of developing mood and anxiety disorders [3]. When left unmanaged, the consequences of ADHD range from impaired quality of life to risk of suicide [4].

While stimulants, including methylphenidate and d-amphetamines, are considered first-line therapeutics for ADHD [5–9], they are associated with adverse reactions such as decreased appetite, headaches, and sleep disturbances [10] and have high abuse liability [11]. This has fueled the development of non-stimulants as ADHD therapeutics [12]. Non-stimulants, such as atomoxetine, guanfacine, clonidine, and viloxazine [13] are considered second-line ADHD therapeutics. Non-stimulants are also commonly associated with adverse events such as fatigue, somnolence, agitation, and aggression and they tend to be less effective than stimulants [14]. Importantly, about one-quarter of patients do not benefit from treatment with neither stimulants nor non-stimulants [7, 15]. Thus, there is an urgent need to develop novel therapeutics for ADHD.

Family, sibling, and adoption studies suggest strong heritability for ADHD [16, 17] and hundreds of relevant genetic markers have been revealed using candidate gene association, genome-wide association, and copy number variant studies [18]. Of the genes identified, *LPHN3* has been most robustly linked to ADHD [19–22]. The *ADGRL3* gene codes for an adhesion G protein-coupled receptor; it has an important role in plasticity, cell adhesion, and synapse formation and is expressed in key brain regions involved with attention [23, 24]. Variants in *ADGRL3* are statistically associated with increased risk of ADHD [25]. This association was replicated in a case-control association study [26]. The *ADGRL3* risk haplotype significantly affects neural function [27] and has been shown to cause both hyperactivity and cognitive deficits [24, 28] in rats and mice [29]. Similarly, studies in larval zebrafish have demonstrated that morpholino oligonucleotide knockdown of *adgrl3.1* function leads to a hyperactive and impulsive motor phenotype [30]. The motor phenotype observed in *adgrl3.1* morphants was rescued by treatment with methylphenidate and atomoxetine [30]. Biological validation of mutations in *ADRGL3* from humans to rodent and zebrafish demonstrates the usefulness of these translational models in ADHD drug discovery [31–33].

The aim of this study was to screen FDA-approved compounds for therapeutic effects in ADHD. To that end, we introduce a novel CRISPR-cas9 *adgrl3.1* mutant model of ADHD in zebrafish and benchmarked the ensuing drug screen against the non-stimulant atomoxetine [30]. First, a simple behavioral phenotype generated by atomoxetine was characterized in wild-type larvae. Next, we screened 1200 compounds for a matching phenotype and re-assayed the hits in the *adgrl3.1* mutant model. Five novel ADHD candidate therapeutics were identified and one was also shown to match the effects of atomoxetine in a cognitive mouse assay.

## Methods and materials

### Fish

For behavioral analysis, a total of *N* = 12480 6 days-post-fertilization (dpf) mutant and *N* = 56528 wild-type larvae were used (all group sizes are reported in supplementary tables 1–6). Wildtype zebrafish (AB line) were initially obtained from ZIRC (Eugene, OR, USA). All zebrafish were maintained in the laboratory at the University of Reykjavik. Zebrafish were fed three times a day on a variable diet of TetraMin flakes (Tetra Holding GmbH, Melle, Germany), Adult Zebrafish Complete Diet (Zeigler Bros, Gardners, PA, USA), and live Artemia (INVE Aquaculture, Incorporation, Salt Lake City, UT, USA). Fish were kept in a 14:10 light:dark cycle (lights-on at 8:00 am) in 10 L multi-tank constant flow system tanks (Aquatic Habitats, Apopka, FL, USA). Water temperature was held at a constant of 28.5 °C and replaced at a rate of 10% per day. All procedures in this study were carried out in strict compliance with the regulations of and approved by, the National Bioethics Committee of Iceland (regulation 460/2017).

### Generation of *adgrl3.1* zebrafish knock out using CRISPR/Cas9

The *adgrl3.1* mutant line (*adgrl3.1*^−/−^ are referred to as homozygous mutants and *adgrl3.1*^+/+^ as WT) was generated as described in [34]. A short guide RNA (sgRNA) was designed containing a PAM motif targeting all the splice variants of *adgrl3.1* shown in the ENSEMBL database: Adgrl3.1-201 exon 11 to 13; Adgrl3.1-202 exon 10 to 12; Adgrl3.1-203 exon 5 to 7; Adgrl3.1-204 exon 9 to 11; Adgrl3.1-205 exon 11 to 13; Adgrl3.1-206 exon 11 to 13. The sgRNA sequences that we used were sgRNA-1: GAGTCCTCCAGTCTGATAGG and sgRNA-2: GCAAGAAGTGTGGGTGCGGT.

**Fig. 1:**
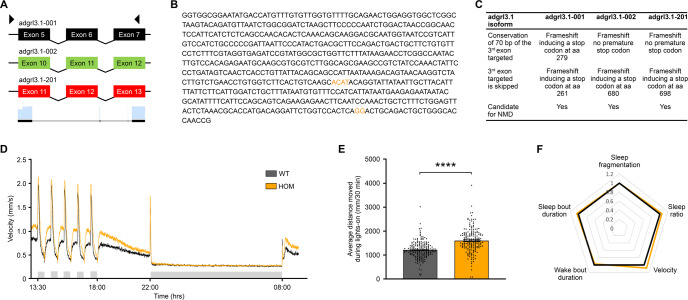
CRISPR-Cas9 mutagenesis and behavioral parameters of *adgrl3.1*^−/−^ larvae. **A** Cartoon showing position of forward and reverse sgRNA constructs used to create the novel *adgrl3.1*^−/−^ line. **B** The base pair sequence of *adgrl3.1* that is deleted by sgRNA injection. The nucleotides shown in orange were not removed and so are retained in the mutant version of *adgrl3.1*. **C** The CRISPR guide RNAs were designed to target the different splice variants of *adgrl3.1* found in the Ensembl database. (NMD: non-sense mediated decay). **D** Swim velocity of homozygous wild type (WT, *n* = 184) and homozygous *adgrl3.1*^−/−^ (HOM, *n* = 179) larvae throughout the whole recording period. The data are pooled from four experiments. The time between 13:30 and 18:00 shows alternating intervals of 30-min lights-off (grey shaded bar) and lights-on phases, followed by constant light-on from 18:00 to 22:00. For the night time, from 22:00 to 08:00 the next morning, the lights were turned off (grey shaded bar). **E** The average distance moved for the five 30-min lights-off periods demonstrates that homozygous *adgrl3.1*^−/−^ larvae moved significantly more than wild-type larvae. **F** Sleep parameters, expressed as fold change of wild-type larvae, were examined throughout the night, during 10-h lights-off phase. No significant differences between genotypes were observed in any of the five sleep parameters. * denote significant differences.

The gRNA constructs were co-injected at a concentration of 100 ng/µl together with 50 ng/nl Cas9 protein (Fig. 1A–C).

We screened for founder fish by in-crossing putative founders and collecting the eggs. Genomic DNA was extracted from these eggs by phenol-chloroform extraction and re-suspended in distilled water. We used the following primers to amplify a fragment of the *adgrl3.1* gene: Forward TGTGCTTTGGTGTGGGTGCTAATGTA and Reverse GGATGCAAAACAGGGTGGGTTGAGGG. 1 µl of 30 ng/µl genomic DNA was used as template in a PCR reaction together with 18.9 µl dH2O, 4 µl Phusion HF buffer, 0.4 µl 10 mM dNTP mix, 0.5 µl primer mix (30 ng/µl each) and 0.2 µl Phusion polymerase. The PCR program used the following parameters: 98 °C 3 min; 40 cycles of [98 °C 30 s; 68 °C 30 s; 72 °C 1.30 min]; 72 °C 5 min and 7 °C hold. The completed PCR reactions were loaded onto a 1% agarose gel containing 5 µl Tris-Borate-EDTA (TBE) in a 100 ml gel. Mutations were identified by a shift in the size of the band produced by PCR on the agarose gel. Promising founders were grown up to adulthood and the nature of the deletion identified by sequencing. The novel *adgrl3.1* mutant line was generated by selecting a founder fish containing a 650 bp deletion that leads frameshift mutations in each splice variant of *adgrl3.1*.

### Genotyping

*adgrl3.1*^+/-^ carriers were identified by fin clipping potential carriers and extracting DNA by either phenol-chloroform extraction or using the HotShot method. A fragment of the *adgrl3.1* gene was amplified using the protocol above, and mutations identified by either gel electrophoresis or Sanger sequencing.

### Behavioral Recordings

Behavioral assays were performed using a custom-built activity monitoring system, as previously described [35, 36]. At 5 dpf, larvae were individually placed in 96-microwell plates (Nunc, Roskilde, Denmark) in system water and acclimated in the system for 24 h. All recordings started at 13:00 with the lights on and continued with alternating dark and light phases in 30-min intervals between 13:30 and 18:00 for the *adgrl3.1* (−/− and +/+) strain but with uninterrupted 14:10 light-dark cycles for wild-types. The behavior was tracked at 5 Hz and larvae that tracked <90% of the total recording time were excluded; each larvae received only one drug/dose

### Motor Assay

Three behavioral parameters were calculated: Average velocity during day and night. Peak velocity (mm/s), defined as the average highest velocity of the five light-to-dark transitions, measured for a 30 s period immediately following the change from light to dark. And distance moved (mm), defined as the average distance moved during the five 30-min light phases.

### Sleep assay

Sleep behavior [37] was recorded in a 96-well plate and analyzed during the lights-off period (22:00 - 08:00). All procedures have been described previously [36, 38, 39]. Briefly, behavioral states were dichotomized into 1-s bins of movement/non-movement (0.5 cm/s set as the threshold for movement). All activity that was slower than that threshold was described as non-movement. After six or more consecutive 1-s bins of non-movement, the seventh second and above were classified as sleep; all other bouts were classified as wake.

### Drug preparation and administration

All compounds were dissolved in dimethyl sulfoxide (DMSO) and subsequently diluted to reach a final concentration between 0.1 and 100 µM in the system water, using 0.3% DMSO as control. Wild-type larvae were assayed using the 1200 compound Prestwick Chemical Library® (Prestwick Chemical, Strasbourg, France) in three concentrations 1, 10, and 35 µM. Mutant larvae were re-assayed using a 67-compound subset of the library in 1, 10, and 30 µM concentrations. Five compounds: aceclofenac, amlodipine, doxazosin, moxonidine (Sigma-Aldrich, St. Louis, MO, USA), and LNP599 (Greenpharma, Orléans, France) were repeated in five concentrations 0.1, 1, 10, 30, and 100 µM. All compounds were prepared the day before recording and administered into the wells between 11:30 and 12:30 on the day of recording.

### Mice

Seventy (male only, 30.6 ± 0.2 g) CD-1 mice (Janvier Labs, Le Genest Saint Isle, France) aged 4-5 weeks were used. The study was conducted with the approval of the Institutional Animal Care and Use Committees (CREMEAS (CEEA 35), Strasbourg, France) and in compliance with European legislation on animal care and scientific experimentation (Permit no: 16808-2018092015164989v3). Mice were group-housed in an enriched environment (sizzle dry, tunnel, and smart house) and maintained in a room with controlled temperature (21–22 °C) and a reversed light-dark cycle (12 h/12 h; lights on: 17:30–05:30; lights off: 05:30–17:30) with food and water available *ad libitum*.

### T-maze spontaneous alternation assay

T-maze spontaneous alternation is an established assay to assess cognitive performance in rodents [40]. The alternation performance is drastically reduced with administration of drugs such as scopolamine and this effect is reversible with cognitive-enhancing [41, 42] drugs. The T-maze assay and apparatus has been described previously [43]. Seven groups of mice (n = 10) were used: (1) Vehicle + Saline; (2) Vehicle + Scopolamine; (3) Scopolamine + Donepezil (0.3 mg/kg); (4) Scopolamine + Atomoxetine (3 mg/kg); (5) Scopolamine + Moxonidine (0.1 mg/kg); (6) Scopolamine + Moxonidine (0.3 mg/kg) and (7) Scopolamine + Moxonidine (1 mg/kg). Scopolamine, Atomoxetine, and Donepezil were used at 1, 3, and 0.3 mg/kg, respectively. All compounds were dissolved in saline and were administered with scopolamine using i.p. injection 30 min prior to the assay except for donepezil which was administrated p.o. 60 min prior to assay. (Scopolamine and Atomoxetine: Sigma-Aldrich, Saint-Quentin-Fallavier, France; Donepezil: Tocris Biotechene, Noyal Chatillon sur Seiche, France).

### Data analysis

Data was obtained using EthoVision XT (Version 11.5.2016, Noldus) and exported to Microsoft Excel for motility analysis and custom-written software for sleep analysis. Statistical analysis was performed using GraphPad Prism Software (Version 8.4.3, GraphPad Software Inc.). For behavioral analysis of zebrafish, statistical differences were evaluated using an unpaired *t*-test and a Bonferroni correction between wild-type and homozygous *adgrl3.1*^−/−^ mutant larvae for all parameters. Statistical differences between compound-treated groups were evaluated using one-way ANOVA and Dunnett’s multiple comparison post hoc analysis. *P* < 0.05 was considered statistically significant. The percentage of spontaneous alternations was calculated as the number of spontaneous alternations divided by 14 (number of free-choice trials). Data was then analyzed using unpaired t-test to evaluate the difference between vehicle + saline and vehicle-+ scopolamine groups and one-way ANOVA with Dunnett´s multiple comparison post hoc test to evaluate the difference between vehicle + scopolamine and the different compound + scopolamine groups. All data are presented as mean ± standard error (s.e.m).

## Results

### *adgrl3.1* mutants exhibit robust hyperactivity phenotype compared to wild-type larvae

*adgrl3.1*^−/−^ larvae were consistently hyperactive compared to their wild type controls while their sleep patterns did not differ (Fig. 1D). Both the average velocity during day (0.874 ± 0.035 mm/s vs. 0.690 ± 0.027 mm/s, *t*(360) = 4.148, *p* < 0.001), and the peak velocity was higher following an abrupt day-time lights-off stimulus (1.632 ± 0.047 mm/s vs. 1.445 ± 0.045 mm/s, *t*(360) = 2.883, *p* < 0.01). However, the velocity during night did not differ (n.s.). The difference in average velocity re-emerged after lights-on (08:00); (0.672 ± 0.032 mm/s vs. 0.535 ± 0.024 mm/s, *t*(360) = 3.454, *p* < 0.001)(Fig. 1E). An independent samples t-test revealed a significant difference between genotypes for the average distance moved during the day-time 30-min lights-on bouts, where *adgrl3.1* homozygous mutant larvae moved significantly more (1590 ± 38.25 vs. 1204 ± 26.75 mm/30 min, *t*(361) = 8.298, *p* < 0.001) than the wild type (Fig. 1F).

Statistical analysis did not reveal a difference between genotypes for any of the five sleep variables (Fig. 1F). We conclude that the average distance moved during daytime 30-min lights-on bouts (hereafter average distance moved; Cohen´s d equals 0.87) is a robust parameter that differentiates well between mutants and wild-types and represents an ideal parameter for drug screening.

### Atomoxetine, clonidine, and guanfacine rescue the motility phenotype of *adgrl3.1* mutants and interfere with sleep parameters

**Fig. 2:**
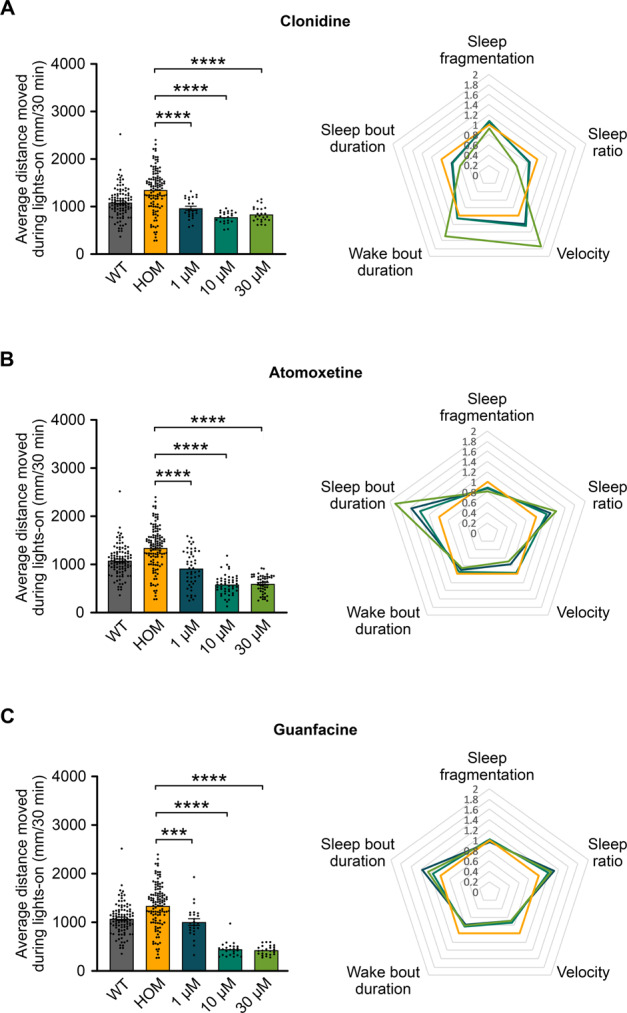
Effects of ADHD therapeutics on *adgrl3.1*^−/−^ mutants. *adgrl3.1*^−/−^ larvae were treated with three different concentrations (1 µM, 10 µM, and 30 µM) of **A** clonidine, **B** atomoxetine, and **C** guanfacine and compared to larvae treated with 0.3% DMSO. Average distance moved during the five 30-min periods of lights-on and sleep parameters during the night was analyzed. Clonidine, atomoxetine and guanfacine all reduced the distance moved during lights-on significantly for all three concentrations. Clonidine, atomoxetine, and guanfacine all significantly altered the sleep dynamics at multiple parameters during the night. Wild-type (WT) larvae treated with 0.3% DMSO are included for visual comparison. * denote significant differences.

To assess the validity of the hyperactive *adgrl3.1*^−/−^ zebrafish model, we assessed the effects of three non-stimulant ADHD therapeutics on the average distance moved and sleep (Fig. 2A–D). One-way ANOVA revealed a statistically significant difference between *adgrl3.1*^−/−^ control (DMSO) group and *adgrl3.1*^−/−^ larvae treated with clonidine (*F*(3, 186) = 26.46, *p* < 0.001), atomoxetine (*F*(3, 258) = 76.36, *p* < 0.001) and guanfacine (*F*(3, 187) = 63.65, *p* < 0.001) for the average distance moved. Dunnett´s post hoc test revealed that all three drugs, lowered the average distance moved for all concentrations tested, 1 µM, 10 µM, and 30 µM, compared to the control group (Supplementary Table 1). In brief, all ADHD therapeutics tested rescued the motility phenotype of the mutant larvae.

All three drugs affected sleep parameters of compound-treated larvae compared to *adgrl3.1*^−/−^ control group (Fig. 2A–C). One-way ANOVA revealed that clonidine had a significant effect on all five sleep parameters, sleep fragmentation (*F*(3,185) = 14.342, *p* < 0.001), sleep ratio (*F*(3,185) = 32.65, *p* < 0.001), velocity (*F*(3,185) = 84.397, *p* < 0.001), wake bout duration (*F*(3,185) = 40.562, *p* < 0.001) and sleep bout duration (*F*(3,185) = 24.443, *p* < 0.001). Atomoxetine: sleep fragmentation (*F*(3,255) = 25.208, *p* < 0.001), sleep ratio (*F*(3,255) = 32.237, *p* < 0.001), velocity (*F*(3,255) = 17.464, *p* < 0.001), wake bout duration (*F*(3, 255) = 4.829, *p* < 0.001) and sleep bout duration (*F*(3, 255) = 26.296, *p* < 0.001). Guanfacine: (*F*(3,185) = 25.531, *p* < 0.001), velocity (*F*(3,185) = 37.473, *p* < 0.001), wake bout duration (*F*(3,185) = 31.125, *p* < 0.001) and sleep bout duration (*F*(3,185) = 16.337, *p* < 0.001) but not sleep fragmentation. Clonidine reduced sleep bout duration and sleep ratio significantly at all three concentrations, and increased sleep fragmentation and velocity significantly at all three concentrations as well as wake bout duration at the highest concentration. Conversely, atomoxetine and guanfacine increased sleep bout duration and sleep ratio significantly at all three concentrations. Sleep fragmentation decreased significantly at all concentrations when treated with atomoxetine but was not affected by guanfacine. Velocity and wake bout duration decreased significantly for all concentrations of guanfacine but only partially for atomoxetine (Supplementary Table 2).

### Triage of the Prestwick chemical library

To narrow possible candidates for drug screening in the mutant model, a simple “behavioral fingerprint” [44, 45] for atomoxetine, the most potent compound tested in a prior study [30], was determined in wild-type larvae using sleep parameters. Next, the 1200 compound Prestwick Chemical Library® was screened for matching effects (three concentrations; 16 naïve control groups; 66 DMSO control groups; *n* = 16; no replicates). The average sleep parameter values were: sleep ratio (59.0 ± 2.32), sleep fragmentation (131.22 ± 4.21), velocity (0.17 ± 0.007 mm/sec), average wake bout duration (10.90 ± 0.39 s) and average sleep bout duration (16.71 ± 1.65 s). Atomoxetine increased fragmentation (up to 145.33 ± 6.12 at the highest dose) and reduced sleep percentage to a range of 29.1–34.4 depending on the dose. Sleep and wake bout durations and velocity were not affected. We thus applied the simplified criterion of a reduction of sleep percentage to less than 40% and increase of fragmentation of 5% to all 1200 compounds.

### Aceloclofenac, amlodipine, doxazosin, and moxonidine rescue motility phenotype of *adgrl3.1* mutants and interfere with sleep parameters

Sixty-seven compounds met the criterion (above) and were re-assayed using the *adgrl3.1*^−/−^ mutant larvae (three concentrations; 5 naïve control groups; 5 DMSO control groups; *n* = 24; no replicates). Four compounds rescued the phenotype.

**Fig. 3:**
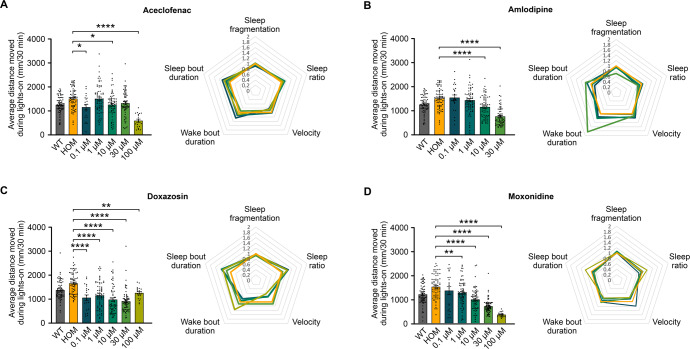
Effects of putative ADHD therapeutics on *adgrl3.1*^−/−^ mutants. *adgrl3.1*^−/−^ larvae were treated with five different concentrations (0.1 µM, 1 µM, 10 µM, 30 µM, and 100 µM) of **A** aceclofenac, **B** amlodipine, **C** doxazosin and **D** moxonidine and compared to larvae treated with 0.3% DMSO. Average distance moved during the five 30-min periods of lights-on and sleep parameters during the night were analyzed. Aceclofenac, amlodipine, doxazosin and moxonidine all reduced the distance moved during lights-on significantly for various concentrations. Aceclofenac, amlodipine, doxazosin and moxonidine all significantly altered the sleep dynamics at multiple parameters during the night. Wild-type (WT) larvae treated with 0.3% DMSO are included for visual comparison. * denote significant differences.

One-way ANOVA revealed a statistically significant difference between *adgrl3.1* control and larvae treated with aceclofenac (*F*(5, 301) = 12.53, *p* < 0.001), amlodipine (*F*(4, 292) = 26.95, *p* < 0.001), doxazosin (*F*(5, 304) = 19.28, *p* < 0.001) and moxonidine (*F*(5, 321) = 38.97, *p* < 0.001) for average distance moved (Fig. 3A–D). Dunnett´s post hoc test revealed that moxonidine lowered the average distance moved in a dose dependent manner for 1, 10, 30, and 100 µM doses (Fig. 3D) and amlodipine at 10 and 30 µM doses (Fig. 3B). Aceclofenac lowered the average distance moved for 0.1, 10, and 100 µM doses (Fig. 3A) and doxazosin for all five doses (Fig. 3C) (Supplementary Table 3). The group exposed to 100 µM doxazosin became non-responsive to stimuli a few hours after drug exposure, and 100 µM dose of amlodipine was lethal.

All four drugs did have an effect on the sleep parameters, but to a lesser extent than the non-stimulant ADHD therapeutics tested (Fig. 3). One-way ANOVA revealed that aceclofenac did have a significant effect on the larval sleep fragmentation (*F*(5,299) = 3.912, *p* < 0.01) and velocity (*F*(5,299) = 4.492, *p* < 0.001) compared to the *adgrl3.1*^−/−^ homozygous control group, but no significant differences were found for sleep ratio, wake bout duration or sleep bout duration (Fig. 3A). Amlodipine had a significant effect on sleep fragmentation (*F*(4,288) = 36.845, *p* < 0.001), sleep ratio (*F*(4,288) = 4.154, *p* < 0.01), wake bout duration (*F*(4,288) = 12.067, *p* < 0.001) and sleep bout duration (*F*(4,288) = 3.535, *p* < 0.01) (Fig. 3B), but no significant differences were found for velocity. Doxazosin did have a significant effect on sleep fragmentation (*F*(5,301) = 2.426, *p* < 0.05), sleep ratio (*F*(5,301) = 6.337, *p* < 0.001), velocity (*F*(5,301) = 27.233, *p* < 0.001) and sleep bout duration (*F*(5,301) = 3.274, *p* < 0.01) compared to the *adgrl3.1*^−/−^ homozygous control group (Fig. 3C), but no significant differences were found for wake bout duration. Lastly, moxonidine had a significant effect on the larvae´s sleep ratio (*F*(5,306) = 3.325, *p* < 0.01), velocity (*F*(5,306) = 10.986, *p* < 0.001) and sleep bout duration (*F*(5,306) = 2.53. *p* < 0.05) (Fig. 3D), but no significant differences were found for sleep fragmentation or wake bout duration. Dunnetts post hoc analysis revealed that significant differences were found between the *adgrl3.1*^−/−^ homozygous control group and larvae exposed to 0.1 µM and 1 µM aceclofenac, 30 µM amlodipine, 10 µM and 30 µM doxazosin and 10 µM moxonidine for sleep fragmentation. Of these four compounds moxonidine administration resulted in the strongest rescue of the motility phenotype while having the least interference with sleep parameters. Dunnett´s post hoc analysis revealed that the only significant difference for sleep parameters at the dose effective for rescuing the behavioral phenotype, was for sleep fragmentation at 10 µM (Supplementary Table 4).

### Moxonidine rescues cognitive deficits in a rodent spontaneous alternation assay

**Fig. 4:**
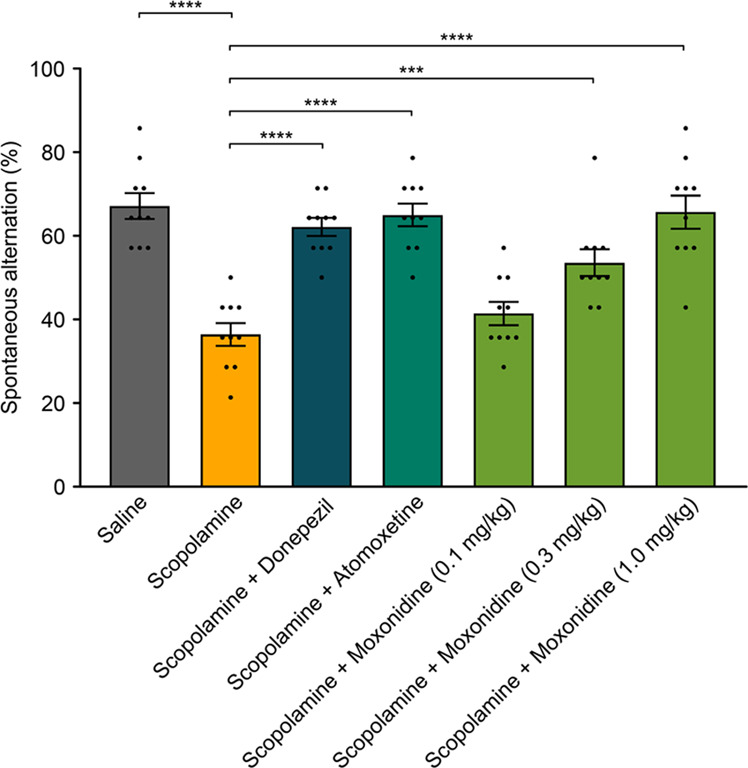
Effects of moxonidine in spontaneous alternation rodent assay. **A** significant difference was found between mice exposed to scopolamine and naïve mice (saline) in which scopolamine-exposed mice showed decrease in spontaneous alterations. Treatment with donepezil and atomoxetine rescued this effect. Moxonidine also rescued this effect in mice in a dose-dependent manner, showing significantly higher spontaneous alterations in mice treated with 0.3 mg/kg and 1 mg/kg treatment but no significant differences were observed between mice treated with 0.1 mg/kg moxonidine compared to scopolamine treated mice. * denote significant differences.

We show that moxonidine also matches the effects of atomoxetine in the spontaneous alternation assay. This is important since this demonstrates efficacy in a different model system and in a different, cognitive, modality. Unpaired *t*-test revealed that vehicle/scopolamine group showed significant reduction in spontaneous alternations as compared to vehicle/saline/group (67% ± 3 vs. 36% ± 2, *t*(18) = 7.521, *p* < 0.001). This decrease in spontaneous alternations reflects cognitive impairment induced by scopolamine. Donepezil and atomoxetine treatment significantly increased (62% ± 2) the spontaneous alternation of scopolamine-mice by 62% ± 2 and 65% ± 3, respectively. Moxonidine treatment resulted in a dose-dependent increase in the spontaneous alternation (41% ± 3, 54% ± 3 and 66% ± 4 for 0.1 mg/kg; 0.3 mg/kg; 1 mg/kg, respectively). The effect was statistically significant (*p* < 0.001) for the 0.3 and 1 mg/kg doses compared to vehicle (Fig. 4).

### LNP599 rescues the motility phenotype of *adgrl3.1*^−/−^ mutants and interferes with sleep parameters

**Fig. 5:**
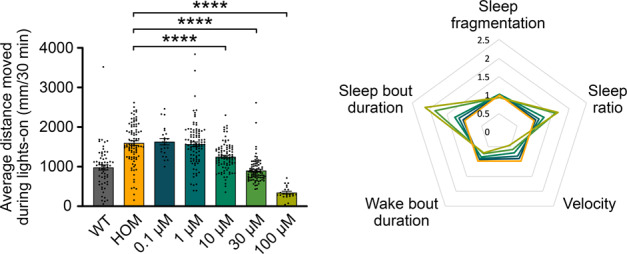
Motility and sleep effects of I1 receptor agonist LNP599. *adgrl3.1*^−/−^ larvae were treated with five different concentrations (0.1 µM, 1 µM, 10 µM, 30 µM, and 100 µM) of LNP599 and compared to larvae treated with 0.3% DMSO. Average distance moved during the five 30-min periods of lights-on and sleep parameters during the night were analyzed. LNP599 reduced the distance moved during lights-on significantly in a dose dependent manner for 10 µM, 30 µM, and 100 µM. LNP599 significantly altered all sleep parameters at various concentrations. Wild type (WT) larvae treated with DMSO are included for visual comparison. * denote significant differences.

Since moxonidine is a potent I1 agonist, we next assayed a pure I1 receptor agonist, LNP599 [46]. One-way ANOVA revealed a significant difference between *adgrl3.1*^−/−^ control and LNP599 treated larvae (*F*(5, 412) = 50.07, *p* < 0.001) for average distance moved (Fig. 5). Dunnett´s post hoc test revealed that LNP599 lowered the average distance moved in a dose dependent manner (Supplementary Table 5). All sleep parameters, sleep fragmentation (*F*(5,408) = 5.825, *p* < 0.001), sleep ratio (*F*(5,408) = 27.464, *p* < 0.001), velocity (*F*(5,408) = 32.004, *p* < 0.001), wake bout durations (*F*(5.408) = 7.781, *p* < 0.001) and sleep bout durations (*F*(5,408) = 21.474, *p* < 0.001) were significantly affected. Sleep fragmentation decreased at 30 µM; sleep ratio increased at 10, 30, and 100 µM; velocity decreased at 10, 30, and 100 µM; wake bout durations decreased at 10, 30, and 100 µM and sleep bout durations increased at 30 and 100 µM (Supplementary Table 6).

## Discussion

In this study we have characterized a novel *adgrl3.1*^−/−^ mutant line, and shown that homozygous *adgrl3.1*^−/−^ mutant larvae are hyperactive compared to their wild-type controls during the light phase. The velocity, however, does not differ during lights-off and neither do their sleep patterns. The model thus fails to capture the sleep disturbances commonly seen in ADHD [47] and, thus leaves no room for therapeutic-driven sleep improvement. However, the model does exhibit the core hyperactive feature of ADHD and is amenable to drug screening [32, 33]. The hyperactive motility phenotype was rescued by atomoxetine, clonidine and guanfacine but with significant impact on the larval sleep profile, consistent with the sleep-promoting properties of α-2 agonists [48] and effects of non-stimulants on sleep in ADHD patients [49]. Four novel compounds rescued the behavioral phenotype of the *adgrl3.1*^−/−^ mutant model: Aceclofenac, amlodipine, doxasozin and moxonidine. Moxonidine, which had the fewest off-target effects on sleep, was also tested in a rodent model of scopolamine-induced cognitive impairment and was subsequently shown to rescue the deficit to the same extent as both donepezil and atomoxetine, further demonstrating cognitive effects of the compound and establishing similar effects as atomoxetine. Moxonidine, has a 30:1 affinity for I1 receptors over α2 adrenoceptors, which inspired us to test a pure I1 agonist, LNP599 [46] that rescued the behavioral phenotype.

The neural substrates that underpin ADHD are not fully elucidated. However, neuroimaging and functional studies have revealed structural and functional differences between individuals with ADHD and controls [17, 50]. In terms of neurobiology, ADHD is conceptualized as a network disorder encompassing neurochemical dysregulation, in particular at the level of the prefrontal cortex that is involved in executive function and attention [51–53]. The anterior cingulate, prefrontal, and orbitofrontal cortices, linked to inattention, hyperactivity, and impulsivity, are interconnected with other cortical and subcortical areas that regulate behavior and emotion. These areas are innervated by DA, NE, and 5-HT projections from the brainstem, and enhanced transmission of these catecholamines represent the major target of ADHD therapeutics [54]. Here we identify potential therapeutics that only partially overlap with known targets.

In general, the current ADHD therapeutics modulate catecholamine neurotransmission, which raises the question about how the novel candidates that rescue the phenotype of the *adgrl3.1*^−/−^ mutant zebrafish function. Aceclofenac is a non-steroidal anti-inflammatory drug and is a non-selective inhibitor of cyclooxygenase (COX; prostaglandin-endoperoxidase synthase) [55]. COX breaks down arachidonic acid to prostaglandin H2 which in turn is catalyzed to prostaglandin D2 by prostaglandin D synthase (PTGDS) [55, 56]. The *PTGDS* gene has higher expression in ADHD patients than bipolar patients and lower mRNA expression in bipolar patients than in healthy controls [57, 58]. *PTGDS* also has a lower expression profile in the spontaneously hypertensive rat (SHR), a classic rodent model of ADHD, compared to control Wistar-Kyoto (WK) rats [59]. Prostaglandin exerts neuromodulatory as well as anti-inflammatory roles and may contribute to the etiology of ADHD, suggesting a role for COX inhibitors, including aceclofenac, in ADHD.

Amlodipine (a dihydropyridine) is an L-type calcium channel (LTCC) blocker [60]. LTCC, Ca_v_1.2 and Ca_v_1.3, encoded by *CACNA1C* and *CACNA1D* respectively, are important regulators of calcium influx to neurons and are critical for normal brain development, function and plasticity [61, 62]. *CACNA1C* and *CACNA1D* have emerged as highly reproducible candidate risk genes for a variety of neuropsychiatric disorders including ADHD [63]. LTCC´s shape neuronal firing, and are present post-synaptically in signaling complexes where they are poised to regulate activity-dependent transcription by calcium second messenger pathways [64]. Moreover, LTCC´s modulate the release of monoamines and prolong after-hyperpolarization in the prefrontal cortex [64, 65].

Doxasozin (a quinazoline) is a selective α_1_ adrenergic blocker [66]. Animal studies have revealed that NE binds α2 receptors during alertness whereas it binds α1 under highly stressful conditions suggesting opposite roles of these receptor systems [67, 68]. Excessive NE signaling has been implicated in schizophrenia, PTSD and mania, and α1 antagonism has been suggested as the mechanism of action for many antipsychotics [52, 69]. Studies using the SHR rat have demonstrated that functional α1 receptors persist in juvenile and adult rats whereas they are depleted in control WK rats, resulting in higher spontaneous locus coeruleus (LC) neuronal activity that can be abolished with α1 antagonists [70]. Unsurprisingly, gene enrichment analysis demonstrate enrichment of α1 receptor signaling in ADHD [71].

Moxonidine is a α2 adrenergic as well as I1 receptor agonist [72]. The rescue of the phenotype described here could thus be explained by moxonidine´s α2 agonism. However, moxonidine has an affinity of 33:1 in favor of I1 over α2 [73]. The endogenous ligand of I1 is agmatine, a polyamine synthesized from L-arginine that interacts with 5-HT, cholinergic, α2 and NMDA receptors in addition to I1 [74]. Agmatine has been linked to a variety of beneficial neural effects including neuroprotection, reduction of neuropathic pain and rescue of cognitive symptoms in mice models of Alzheimer´s disease, and a reduction in depressive symptoms as well as amelioration of compulsive-like behaviors [75–78]. To our knowledge I1 has not been implicated in ADHD previously. Could the behavioral rescue be mediated via the I1 receptor system? Neurobiologically this is plausible via the medial habenula (MH). The main efferent projections of the medial habenula are to the interpeduncular nucleus (IPN) via the fasciculus retroflexus [79, 80]. The IPN in turn gives rise to ascending projections to limbic structures and rodent studies have revealed that the IPN exerts a tonic inhibition on mesocortical, mesolimbic, and mesostriatal dopaminergic neurons [81]. Furthermore, rodent electrophysiological studies have demonstrated strong MH inhibition by agmatine and moxonidine – an effect that is abolished with efaroxan, an I1 antagonist [82]. In zebrafish, habenular lesions increase anxiety-like behaviors and reduce behavioral adaptations [83, 84]. Therefore, it is plausible that I1-mediated habenular inhibition by moxonidine results in less IPN activity, enhancing tonic mesocortical DA transmission which in turn ameliorates the ADHD-like symptoms of the model tested here.

LNP599 is a little-studied pure I1 agonist [46]. The compound has thus far been shown to improve metabolic syndrome, reduce blood pressure and heart rate and reduce plasma catecholamine levels in rodent models [85–88]. LNP599 also rescues the hyperactive phenotype. The results suggest a role for this receptor system in ADHD that may, at least partially, explain the effects of moxonidine and clonidine.

Before these compounds can be developed into treatments for managing ADHD more assays should be performed including assays for attention and aggression, in addition to verification in a mammalian model. It is noteworthy that three of the compounds have been prescribed for hypertension. Importantly, 14 compounds of the 67-compound subset used for the *adgrl3.1*^−/−^ assay have been prescribed for hypertension, and nine of them fail to show efficacy in the model, showing that the model is not selective for compounds used to treat hypertension.

In the current paper we describe a novel zebrafish model of ADHD and identify five potential therapeutics for the disease; four repurposed and one novel. The results offer a novel tool to study ADHD, offer insights into the neural substrates of the disease, and identify compounds that could be developed into novel therapeutics.

## Supplementary information

Supplementary Tables 1-6
